# Risk factors for recurrent falls in midlife: a prospective cohort study using data from the Health and Employment After Fifty (HEAF) study

**DOI:** 10.1136/bmjopen-2026-118710

**Published:** 2026-05-27

**Authors:** Stefania D’Angelo, Leo Westbury, Faidra Laskou, Karen Walker-Bone, Elaine Dennison

**Affiliations:** 1 MRC Lifecourse Epidemiology Centre, University of Southampton, Southampton, UK; 2 Monash University School of Public Health and Preventive Medicine, Melbourne, Victoria, Australia

**Keywords:** Aging, Public health, Epidemiology

## Abstract

**Abstract:**

**Background:**

Falls, especially recurrent, cause significant morbidity. Research on falls generally focuses on older adults but patterns of falling may start earlier in life. This study aimed to quantify the prevalence of falls and recurrent falls among late middle-aged community-dwelling adults and identify the socio-demographic, lifestyle and health-related factors associated with recurrent falls.

**Methods:**

The Health and Employment After Fifty (HEAF) study is a longitudinal cohort of men and women aged 50–64 years recruited in 2013–14 from across England. At baseline and each of five approximately annual follow-ups, participants reported falls in the preceding year. Participants were categorised as recurrent fallers if they experienced more than one fall on at least two occasions, non-fallers if they never reported a fall, or intermediate fallers otherwise. Multinomial logistic regression explored associations between fall category and potential risk factors, presented as relative risk ratios with 95%CI.

**Results:**

Among 8134 participants, 7051 were eligible for this analysis. The prevalence of any falls ranged from 14–18% across follow-ups. Overall, 437 (6%) were recurrent fallers, 2738 (39%) intermediate and 3876 (55%) non-fallers. Independent predictors of recurrent falls included female gender, unpartnered, unemployed or retired and lack of home ownership. Health-related factors included obesity, fair/poor self-rated health, depression, poor sleep, slow walking speed and memory problems. The final model correctly classified 60% of participants.

**Conclusions:**

Recurrent falls in mid-life were relatively common. Both socio-economic and health-related characteristics, alongside female gender, were identified as predictors, suggesting potential targets for early identification and risk mitigation in this age group.

**STRENGTHS AND LIMITATIONS OF THIS STUDY**

- The main strength is the population-based design that ensures generalisability of findings.
- Additionally, the availability of a wide range of sociodemographic, health and lifestyle factors, and the long follow-up period.
- The main limitation is how the outcome was assessed. A more detailed question about the number of falls experienced would have enabled a more nuanced analysis.
- Information on fall severity, consequences and hospitalisation was not collected.

## Introduction

 Falls are a major public health concern and a leading cause of disability, morbidity and mortality.^1 2^ Most research to date has focused on older adults who experience the highest incidence of falls. In a study from the USA, 15% of women and 11% of men aged 55–64 years experienced at least one fall in the previous 12 months.^3^ Similarly, a survey of Australian women aged 50–55 years conducted in 2010 found that up to 30% reported a fall in the previous year.^4^ Falls among late middle-aged adults appear to be relatively common yet remain comparatively understudied.^5 6^

Importantly, there has been limited research quantifying the prevalence of recurrent falls (ie, reporting more than one fall^7^ over a certain period) in this age group. One study among community-dwelling women aged 40–60 years in Sri-Lanka reported a recurrent fall prevalence of 13%,^8^ while age-standardised prevalence of recurrent falls was 4.6% in an ethnically diverse Asian population aged 40 and older.^9^

Recurrent falls are clinically important because individuals who fall repeatedly tend to experience more severe consequences, including higher rate of injury, functional decline, healthcare utilisation and mortality.^1 10^ Patterns of falling may be established at younger ages than traditionally researched, but the prevalence and determinants of recurrent falls in late middle-aged adults are under-studied. A systematic review and meta-analysis of 22 cohort studies suggested that among older adults aged 60 or older, markers of frailty were strongly associated with the risk for recurrent falls.^11^

Identifying risk factors associated with recurrent falls in mid-life is important for early recognition of high-risk groups and for informing the development of targeted fall prevention strategies.

Therefore, this study aimed to (1) quantify the prevalence of falls and recurrent falls among late middle-aged community-dwelling adults; (2) identify the sociodemographic, lifestyle and health-related factors associated with recurrent falling.

## Methods

Data were from the Health and Employment After Fifty (HEAF) study. This is a geographically representative, population-based cohort of men and women recruited from around England in 2013–14 when they were aged between 50 and 64 years.^12^ Participants were recruited from 24 general practice surgeries, chosen among those who contributed data to the Clinical Practice Research Datalink (CPRD). The surgeries mailed a consent form and a baseline questionnaire to all individuals born between 1948 and 1962 (other than those suffering from terminal illnesses or recently bereaved). Those who completed baseline questionnaire and returned the written consent form to the HEAF research team were enrolled into the study and were sent annual follow-up questionnaires. At baseline and at each annual follow-up for a total of approximately 6 years, participants reported a variety of socio-demographic, health-behaviours, health and work-related factors. Participants returned a maximum of six questionnaires, including the baseline one. 93% of the sample consented to data linkage with the CPRD, a national database of medical consultations, referrals and medications. The linkage took place in 2015.

### Outcome

In each questionnaire, participants self-reported whether they had experienced any fall in the previous 12 months (0, 1, >1). Participants were categorised as recurrent fallers if they experienced more than one fall on at least two occasions over the course of the study, non-fallers if they never reported a fall over the study duration and intermediate fallers if they reported at least one fall but did not meet the criteria for recurrent fallers.

### Socio-economic, lifestyle and health variables

A panel of risk factors was evaluated in their association with recurrent falls. All risk factors in this analysis were assessed at baseline.

Since falls are multifactorial events, influenced by socio-demographic, lifestyle, physical and behavioural factors, we selected variables across each of these domains.

Socio-demographic factors, such as age, sex, highest educational qualification, marital status (single/widowed/divorced, married/living with partner),^13 14^ as well as indicators of socioeconomic position such as home ownership (rented, mortgaged, owned outright/rent free), self-perceived financial status (at least getting by financially, managing with difficulty), proportion of family income contributed by the participant (≥1/2, between 1/2 and 1/4, less than 1/4)^15 16^ and employment status (employed, self-employed, unemployed, retired) are widely recognised as important determinants of falls. These factors influence exposure to environmental hazards, access to resources and overall vulnerability to adverse health outcomes, including recurrent falls.

Lifestyle factors such as living with obesity (BMI was calculated based on self-reported height and weight and obesity was present if BMI≥30 kg/m^2^),^17^ alcohol consumption,^18^ smoking habits and leisure time physical activity (none, ≤5 hours/week, 5–10 hours/week, >10 hours/week)^19^ can influence balance, mobility and exposure to activity-related hazards. Health-related variables, such as self-rated health (SRH) (fair/poor, at least good), depressive symptoms assessed with the CES-D score (coded as depressed if CES-D≥16), poor sleep, any musculoskeletal pain in the previous 12 months, walking speed (normal/fast vs very slow/unable to walk) and memory problems (no serious problems and not worsened in the past year vs serious problems or a lot worse in the past year) capture functional and clinical characteristics that may indicate underlying health impairments.^20^,^22^ Most variables were re-categorised prior to the analysis to increase statistical power and avoid sparse data issues.

In addition, using CPRD codes, we computed the number of comorbidities for each participant, with any of the five types of conditions: neurological; mental health; hypertension or cardiovascular; respiratory and musculoskeletal (scored 0–5). This score was dichotomised for analysis as: no comorbidities versus at least one.

### Statistical analysis

The sample included in the analysis was described using summary statistics, stratified by categories of falls. Categorical variables are presented as numbers and percentages while continuous normally distributed variables are summarised with means and SD. A non-parametric test for trend was used to assess differences across categories of falls.

Since our main outcome has a natural ordering (non-fallers being the least severe category and recurrent fallers being the most severe one), we initially performed an ordinal logistic regression. However, since the assumption of proportional odds (ie, the effect of an independent variable is constant for each increase in the level of response) was not met in our sample, we opted for a multinomial logistic regression instead. This approach produces two separate estimates, one for the intermediate group and one for the recurrent fallers, each compared with the baseline category of non-fallers. Estimates are presented as relative risk ratios (RRR) with 95% CIs.

Our primary aim was to identify the strongest predictors of repeated falls through a prediction model rather than to make casual inference. We first built minimally adjusted models including age and sex only. Subsequently, a multivariate model was developed by including all variables that were significantly related to repeated falling at the initial stage (p<0.001). Multicollinearity was assessed with the variance inflation factors to ensure that variables included in the final model were not highly correlated. The predicted ability of the final multinomial logistic regression model was evaluated using model-based predicted probability and percentage correct classification. For each individual, the predicted probability of belonging to each outcome category (non-faller, intermediate faller and recurrent faller) was obtained from the fitted model, and the category with the highest predicted probability was taken as the individual’s predicted class. Classification performance was computed by comparing predicted and observed categories to calculate the proportion of individuals correctly classified. In addition, the mean predicted probabilities for each outcome were computed within observed outcome categories to examine how well the model discriminated between faller groups.

Internal validation of the final multivariate model was performed using bootstrap resampling with 1000 replications. Estimates with bootstrapped CIs are reported in online supplemental table 1. In the primary analysis, all participants were included, with missing values assigned to a separate category. A sensitivity analysis was subsequently performed restricting the sample to participants with complete data for all variables included in the multivariate model. All analyses were performed with Stata statistical software v19.5. The reporting of this observational study follows the STROBE checklist.

## Results

**Figure 1:**
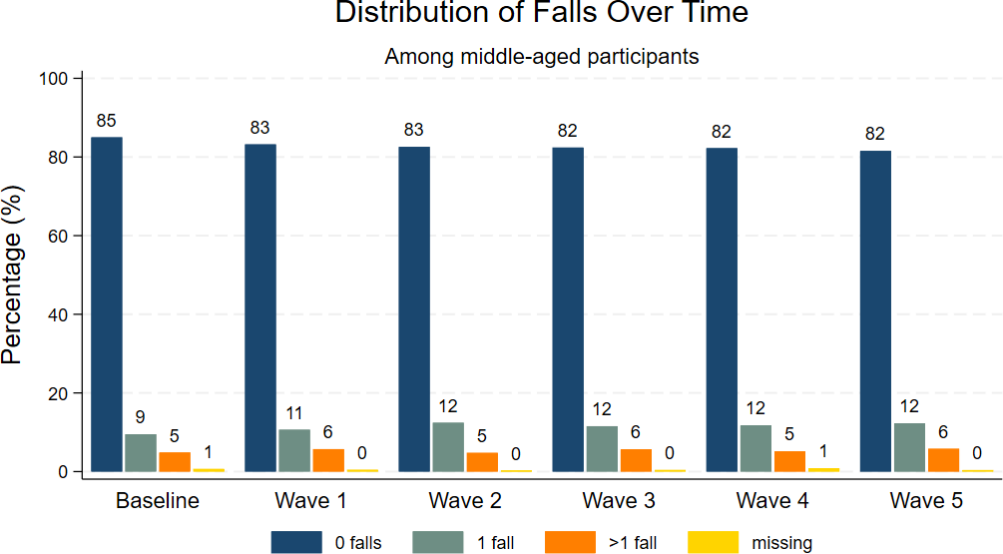
Number of falls reported at each wave of data collection.

Among 8134 participants recruited into the HEAF study, 574 did not consent to CPRD data linkage, and a further 509 were excluded as only baseline data were available which made it impossible to define the outcome, leaving 7051 in the analysis (n=3861, 54.8% females). The number of follow-ups available per person ranged between 1 and 5 (in addition to baseline). 74% of the sample had data on all five follow-ups. The remaining 26% was split into: 6% had four follow-ups; 7% had three follow-ups; 9% had two follow-ups and 4% had data on baseline and one follow-up only. The proportion of study participants reporting at least one fall was stable over time, at approximately 14–18% of the sample at each wave of data collection as reported in figure 1.

A total of 437 participants (6% of the sample) were classified as recurrent fallers, 2738 (39%) as intermediate fallers and 3876 (55%) as non-fallers.

Table 1 highlights several differences in participants’ characteristics across the fall categories. Recurrent fallers were more likely to be women and unpartnered (single, widowed or divorced). They also presented a less favourable socio-economic profile: lower levels of educational attainment, reporting financial difficulties, not homeowners, contributing minimally to household income and not in paid employment. In terms of lifestyle and health behaviours, recurrent fallers were more likely to live with obesity, be current smokers, have low alcohol intake or be a non-drinker and be physically inactive. They also reported poorer health outcomes than other groups, including poorer SRH, more depressive symptoms, poorer sleep, musculoskeletal pain and very slow walking speed. Finally, they were more likely to report at least one comorbidity obtained via data linkage with CPRD.

**Table 1:** Baseline characteristics of the sample, by categories of falls

Baseline characteristic	Non-fallers (n=3876)	Intermediate fallers (n=2738)	Recurrent fallers (n=437)	P value test for trend
Sex, female	1861 (48.0%)	1729 (63.2%)	271 (62.0%)	<0.001
Age, median (IQR)	58.6 (54.6,62.4)	59.4 (55.4,62.9)	58.6 (55.0,62.5)	<0.001
Marital status				
Single/widowed/divorced	1001 (25.8%)	821 (30.0%)	191 (43.7%)	<0.001
Qualification level				<0.001
No qualification/school only	1307 (33.7%)	968 (35.4%)	183 (41.9%)	
Vocational training certificate	1152 (29.7%)	834 (30.5%)	142 (32.5%)	
University degree or higher	1417 (36.6%)	936 (34.2%)	112 (25.6%)	
Perceived financial status				<0.001
At least getting by	3553 (91.7%)	2450 (89.5%)	325 (74.4%)	
Managing with difficulty	266 (6.9%)	239 (8.7%)	104 (23.8%)	
Missing	57 (1.5%)	49 (1.8%)	8 (1.8%)	
Proportion of family income coming from you				<0.001
≥1/2	1935 (49.9%)	1185 (43.3%)	151 (34.6%)	
Between 1/4 and 1/2	530 (13.7%)	396 (14.5%)	44 (10.1%)	
<1/4	1282 (33.1%)	1048 (38.3%)	214 (49.0%)	
Missing	129 (3.3%)	109 (4.0%)	28 (6.4%)	
Home ownership				<0.001
Rented	416 (10.7%)	332 (12.1%)	117 (26.8%)	
Mortgaged	1225 (31.6%)	811 (29.6%)	132 (30.2%)	
Owned outright or rent-free	2178 (56.2%)	1543 (56.4%)	179 (41.0%)	
Missing	57 (1.5%)	52 (1.9%)	9 (2.1%)	
Employment status				<0.001
Employed	2272 (58.6%)	1515 (55.3%)	170 (38.9%)	
Self-employed	507 (13.1%)	311 (11.4%)	38 (8.7%)	
Unemployed	162 (4.2%)	158 (5.8%)	92 (21.1%)	
Retired	935 (24.1%)	754 (27.5%)	137 (31.4%)	
Obesity				<0.001
<30 (non-obese)	3007 (77.6%)	1958 (71.5%)	248 (56.8%)	
≥30 (obese)	782 (20.2%)	695 (25.4%)	169 (38.7%)	
Missing	87 (2.2%)	85 (3.1%)	20 (4.6%)	
Smoking				0.001
Never/ex	3459 (89.2%)	2438 (89.0%)	348 (79.6%)	
Current	390 (10.1%)	263 (9.6%)	81 (18.5%)	
Missing	27 (0.7%)	37 (1.4%)	8 (1.8%)	
Alcohol consumption				<0.001
Low/no drinker	737 (19.0%)	542 (19.8%)	123 (28.2%)	
Moderate drinker	2035 (52.5%)	1443 (52.7%)	167 (38.2%)	
Heavy drinker	777 (20.1%)	466 (17.0%)	66 (15.1%)	
Missing	377 (8.4%)	287 (10.5%)	81 (18.5%)	
Leisure time physical activity				<0.001
None	831 (21.4%)	678 (24.8%)	166 (38.0%)	
≤5 hours/week	2026 (52.3%)	1368 (50.0%)	151 (34.6%)	
5-10 hours/week	715 (18.5%)	465 (17.0%)	53 (12.1%)	
>10 hours/week	207 (5.3%)	158 (5.8%)	35 (8.0%)	
Missing	97 (2.5%)	69 (2.5%)	32 (7.3%)	
Self-rated health				<0.001
At least good	3262 (84.2%)	2025 (74.0%)	161 (36.8%)	
Fair/Poor	575 (14.8%)	657 (24.0%)	268 (61.3%)	
Missing	39 (1.0%)	56 (2.1%)	8 (1.8%)	
Depressive symptoms				<0.001
Not depressed	3189 (82.3%)	1943 (71.0%)	188 (43.0%)	
Depressed	659 (17.0%)	765 (27.9%)	241 (55.2%)	
Missing	28 (0.7%)	30 (1.1%)	8 (1.8%)	
Poor sleep				<0.001
No	3368 (86.9%)	2188 (79.9%)	239 (54.7%)	
Yes	508 (13.1%)	550 (20.1%)	198 (45.3%)	
Any musculoskeletal pain				<0.001
No	3153 (81.4%)	1940 (70.9%)	157 (35.9%)	
Yes	698 (18.0%)	780 (28.5%)	277 (63.4%)	
Missing	25 (0.6%)	18 (0.7%)	3 (0.7%)	
Walking speed				<0.001
Normal/fast	3793 (97.9%)	2541 (92.8%)	281 (64.3%)	
Unable/very slow	81 (2.1%)	171 (6.3%)	153 (35.0%)	
Missing	2 (0.1%)	26 (1.0%)	3 (0.7%)	
Problems with your memory				<0.001
No serious problems & not worsened	3781 (97.6%)	2589 (94.6%)	373 (85.4%)	
Serious problems or a lot worse	79 (2.0%)	104 (3.8%)	55 (12.6%)	
Missing	16 (0.4%)	45 (1.6%)	9 (2.1%)	
CPRD - N. comorbidities				<0.001
None	1513 (39.0%)	921 (33.6%)	75 (17.2%)	
At least one	2363 (61.0%)	1817 (66.4%)	362 (82.4%)	

CPRD, Clinical Practice Research Datalink.

Table 2 shows the sex- and age-adjusted association between each potential risk factor (assessed at baseline) and falling category. Relative risk ratios are presented separately for intermediate fallers and recurrent fallers, with non-fallers as the reference category. Compared with non-fallers, both intermediate and recurrent fallers showed significant associations with multiple sociodemographic, lifestyle and health factors, with stronger and more consistent effects observed among recurrent fallers.

**Table 2:** Association between individual baseline characteristics and fall category, adjusted for sex and age at recruitment

Baseline characteristic	Intermediate fallers (vs Non fallers)		Recurrent fallers (vs Non fallers)	
	RRR (95% CI)	P value	RRR (95% CI)	P value
Sex, Female	1.87 (1.69 to 2.06)	<0.001	1.77 (1.44 to 2.17)	<0.001
Age at recruitment				
55-59 (vs 50–54)	1.24 (1.09 to 1.42)	0.001	1.30 (1.00 to 1.68)	0.05
60-64 (vs 50–54)	1.35 (1.19 to 1.54)	<0.001	1.03 (0.79 to 1.34)	0.82
65-66 (vs 50–54)	1.46 (1.20 to 1.79)	<0.001	1.39 (0.94 to 2.07)	0.10
Marital status				
Single/widowed/divorced	1.20 (1.07 to 1.34)	0.001	2.17 (1.77 to 2.66)	<0.001
Qualification level				
No qualification/school only	1.04 (0.93 to 1.18)	0.47	1.69 (1.32 to 2.17)	<0.001
Vocational training certificate	1.10 (0.97 to 1.24)	0.13	1.57 (1.21 to 2.03)	0.001
University degree or higher	Ref		Ref	
Perceived financial status				
At least getting by	Ref		Ref	
Managing with difficulty	1.36 (1.13 to 1.64)	0.001	4.40 (3.40 to 5.68)	<0.001
Missing	1.20 (0.81 to 1.78)	0.35	1.48 (0.70 to 3.13)	0.31
Proportion of family income coming from you				
≥1/2	Ref		Ref	
Between 1/4 and 1/2	1.07 (0.92 to 1.24)	0.39	0.97 (0.68 to 1.38)	0.86
<1/4	1.05 (0.93 to 1.18)	0.46	2.08 (1.63 to 2.64)	<0.001
Missing	1.14 (0.87 to 1.49)	0.36	2.70 (1.73 to 4.22)	<0.001
Home ownership				
Rented	1.23 (1.04 to 1.44)	0.01	3.67 (2.83 to 4.75)	<0.001
Mortgaged	1.07 (0.96 to 1.21)	0.23	1.46 (1.14 to 1.87)	0.003
Owned outright or rent-free	Ref		Ref	
Missing	1.31 (0.89 to 1.93)	0.17	1.93 (0.94 to 3.97)	0.08
Employment status				
Employed	Ref		Ref	
Self-employed	0.99 (0.84 to 1.16)	0.87	1.10 (0.76 to 1.59)	0.62
Unemployed	1.46 (1.16 to 1.84)	0.001	7.48 (5.54 to 10.11)	<0.001
Retired	0.95 (0.83 to 1.09)	0.50	1.94 (1.46 to 2.57)	<0.001
Obesity				
<30 (non-obese)	Ref		Ref	
≥30 (obese)	1.39 (1.13 to 1.56)	<0.001	2.64 (2.14 to 3.27)	<0.001
Missing	1.48 (1.09 to 2.01)	0.01	2.73 (1.65 to 4.54)	<0.001
Smoking				
Never/ex	Ref		Ref	
Current	1.00 (0.85 to 1.19)	0.97	2.13 (1.64 to 2.78)	<0.001
Missing	1.86 (1.12 to 3.08)	0.02	2.87 (1.29 to 6.39)	0.01
Alcohol consumption				
Low/no drinker	Ref		Ref	
Moderate drinker	1.04 (0.91 to 1.18)	0.60	0.52 (0.40 to 0.67)	<0.001
Heavy drinker	1.12 (0.95 to 1.33)	0.19	0.64 (0.46 to 0.89)	0.009
Missing	1.15 (0.95 to 1.40)	0.15	1.48 (1.08 to 2.01)	0.01
Leisure time physical activity				
None	0.98 (0.77 to 1.23)	0.84	1.07 (0.72 to 1.59)	0.73
≤5 hours/week	0.85 (0.68 to 1.06)	0.16	0.41 (0.28 to 0.62)	<0.001
5-10 hours/week	0.82 (0.64 to 1.04)	0.44	0.42 (0.26 to 0.66)	<0.001
>10 hours/week	Ref		Ref	
Missing	0.93 (0.64 to 1.36)	0.71	1.93 (1.13 to 3.31)	0.02
Self-rated health				
At least good	Ref		Ref	
Fair/Poor	1.90 (1.67 to 2.15)	<0.001	9.77 (7.87 to 12.13)	<0.001
Missing	2.17 (1.43 to 3.29)	<0.001	4.06 (1.86 to 8.86)	<0.001
Depressive symptoms				
Not depressed	Ref		Ref	
Depressed	1.92 (1.71 to 2.17)	<0.001	6.23 (5.05 to 7.69)	<0.001
Missing	1.85 (1.09 to 3.12)	0.02	5.07 (2.27 to 11.31)	<0.001
Poor sleep				
No	Ref		Ref	
Yes	1.61 (1.41 to 1.84)	<0.001	5.33 (4.31 to 6.60)	<0.001
Any musculoskeletal pain				
No	Ref		Ref	
Yes	1.83 (1.62 to 2.05)	<0.001	7.97 (6.44 to 9.87)	<0.001
Missing	1.24 (0.67 to 2.29)	0.50	2.58 (0.77 to 8.66)	0.13
Walking speed				
Normal/Fast	Ref		Ref	
Unable/Very slow	3.13 (2.39 to 4.11)	<0.001	26.70 (19.78 to 36.03)	<0.001
Missing	19.49 (4.60 to 82.62)	<0.001	22.47 (3.72 to 135.65)	0.001
Problems with your memory				
No serious problems & not worsened	Ref		Ref	
Serious problems or a lot worse	1.93 (1.43 to 2.60)	<0.001	7.02 (4.89 to 10.09)	<0.001
Missing	3.92 (2.20 to 6.99)	<0.001	5.62 (2.46 to 12.86)	<0.001
CPRD - N. comorbidities				
None	Ref		Ref	
At least one	1.21 (1.09 to 1.34)	<0.001	3.06 (2.36 to 3.96)	<0.001

CPRD, Clinical Practice Research Datalink; RRR, relative risk ratio.

Female sex was associated with increased risk of both intermediate (RRR 1.87, 95% CI 1.69 to 2.06) and recurrent falling (RRR 1.77, 95% CI 1.44 to 2.17). Being unpartnered increased the risk of both intermediate and recurrent falling, while lower educational level, financial difficulty, renting and unemployment were particularly strong predictors of recurrent falling. Obesity was associated with both intermediate and recurrent falling. Current smoking was associated with recurrent but not intermediate falling. Lower levels of leisure-time physical activity, as well as low or no consumption of alcohol, were also associated with a higher likelihood of recurrent falls. Fair/poor SRH, depressive symptoms, poor sleep, musculoskeletal pain and number of comorbidities from CPRD were all strongly associated with recurrent falling, with smaller but still significant effects for intermediate falling. Walking speed showed the strongest association with recurrent falling, while severe memory problems were also strongly associated.

**Figure 2:**
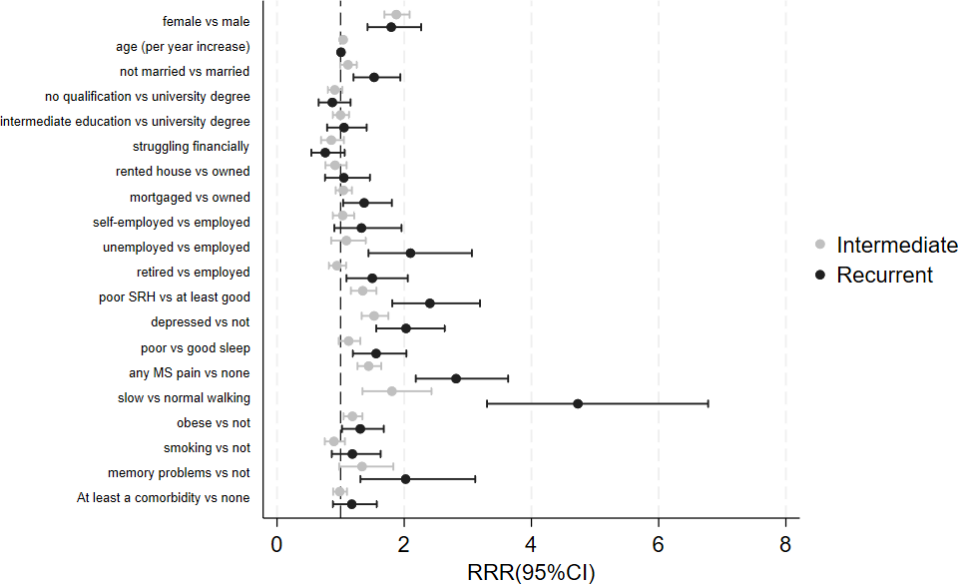
Multivariate model showing association between selected risk factors and fall categories.

Figure 2 shows mutually adjusted estimates from a model including all covariates significant in the sex-and-age-adjusted model. This shows that factors that independently increased the risk of being a recurrent faller (vs non faller) were being a woman, unpartnered, unemployed or retired, and with a mortgage (vs owning home). Additionally, several health-related factors remained significant, such as fair/poor SRH, depression, poor sleep, slow walking speed and memory problems. Most of these factors were also significant for the intermediate fallers category, although associations were generally weaker.

The final multivariate model has an overall classification accuracy of 60%, meaning that, based on the variables included in the model, 60% of the individuals are correctly allocated to one of the three categories of falls. However, mean predicted probabilities by observed category indicate that the model assigns the highest probabilities to the correct outcome for non-fallers (p1=0.59) and intermediate fallers (p2=0.41). For recurrent fallers, the highest mean predicted probability is 0.42 for intermediate falls, rather than 0.23 for recurrent, indicating that the model tends to under-predict this less frequent category.

In a complete case analysis based on 6802 participants with non-missing data on all covariates included in the multivariate model, effect sizes and levels of significance were very similar (data not shown).

## Discussion

In this large cohort of more than 7000 late middle-aged men and women in England, the annual prevalence of falls ranged between 14% and 18%, indicating that falls are relatively common even before older age. Moreover, 6% of the sample experienced recurrent falls, defined as reporting more than one fall at least twice over the 6 years of study period.

Several sociodemographic characteristics were associated with an increased likelihood of recurrent falls compared with not falling at all. Women who were unpartnered and who were unemployed or retired (compared with those in paid employment) had higher risks of recurrent falling. Housing tenure also appeared relevant, with participants with a mortgage showing greater risk than those owning their home outright. Recurrent fallers additionally exhibited multiple indicators of poorer mental and physical health.

Before comparing our findings with existing literature, it is important to consider strengths and limitations of the study. A key strength is the use of a population-based cohort that is reasonably representative of the general population in terms of age, sex, marital status and employment status. All deciles of material deprivation were captured; however, the cohort lacks ethnic diversity, with 98% of it identifying as white, limiting generalisability to more diverse populations.^12^ Another strength is the availability of a wide range of sociodemographic, health lifestyle factors and the long follow-up period.

The main limitation relates to the assessment of the outcome, which limited the way we could define recurrent fallers. A more detailed question, capturing the exact number of falls experienced, would have enabled a more nuanced analysis. As a result, our definition treats a participant who fell twice per year for 2 years in the same way as someone who fell ten times per year for 5 years. This approach therefore does not recognise that the severity of the fall outcome should be regarded as much worse in the latter, and as a consequence, this may have diluted the observed associations. Furthermore, information on fall severity, consequences, whether the fall resulted in hospitalisation and the circumstances surrounding each fall were not collected. The lack of contextual detail limits our ability to distinguish between falls occurring during high-intensity activities and those taking place during lower intensity activities.

The prevalence of falls in the past year in our cohort aligns with estimates from comparable age groups, such as the 15% of women and 11% of men reporting a fall among community-dwelling adults aged 55–64 in the United States.^3^

Our study reinforces the importance of factors such as memory problems, depression, musculoskeletal pain and slow walking speed in predicting repeated falls, consistent with previous evidence.^11^ For example, depressive symptoms were associated with a 76% increase in the odds of recurrent falls in a cohort of 6000 adults aged 40–65 in Japan.^23^ In a longitudinal study among community-dwelling older adults, decreased gait speed over 12 months was associated with a significantly increased risk of multiple falls.^24^ Slow walking speed in healthy adults in their 40s has also been linked to poorer physical and cognitive function and is considered a good indicator of accelerated ageing.^25^

However, our findings regarding the influence of socio-demographic characteristics, including home ownership and employment status, understudied previously, represent a novel contribution to the literature. After accounting for other factors, being retired—compared with being in paid employment—was associated with a 50% increased risk of repeated falls, a finding that mirrors results from a sample of 50–64 year olds participating in the National Survey of Health and Development (NSHD) cohort.^6^ This has important implications suggesting ongoing employment to older ages might convey benefit for reducing falls risk. A previous meta-analysis of 22 longitudinal studies of adults aged 60 years and older highlighted the need for further research into environmental and socio-demographic determinants of recurrent falls.^11^

These findings highlight the importance of socio-economic factors in predicting healthy ageing. Falling as an older adult has massive consequences for fracture risk, mobility, being able to live independently and longevity.^26 27^ Even among adults aged 64 years on average at the end of this study, there were clear socioeconomic determinants of recurrent falls. This suggests that those who have the lowest pension entitlement, who still need to pay rent after retirement and who have most comorbidities are also most vulnerable to the health and mobility consequences associated with recurrent falls. This gives enormous potential impetus to development and implementation of strategies aimed at identifying these individuals early and offering interventions to prevent this, which could be cost saving as well as lifesaving.

In line with other prospective studies linking alcohol consumption and falls hospitalisation^28^ or risk of falls,^29^ in sex- and age-adjusted analyses, moderate or high alcohol consumption, relative to no/low consumption, was associated with a reduced risk of recurrent falls. This potentially reflects residual confounding or reverse causation due to underlying health conditions among non-drinkers. Consistent with our results, a recent meta-analysis of 24 studies concluded that among individuals aged 60 and older, obesity was associated with increased risk of falls and of multiple falls.^30^

Although older age was significantly associated with recurrent falls in univariate analyses, this association attenuated after adjustment for other factors. This contrasts with findings from previous studies that have reported fall risk to increase with age, although this may reflect the narrow age range of the sample. For example, a multi-country population-based cohort study found fall risk to rise steadily with age,^6^ as did a study among middle-aged Indian women, in which age remained a strong predictor of recurrent falls risk.^8^ Similarly, a US cross-sectional study observed increasing fall frequency with age among both men and women.^31^ One possibility is that the age range in our sample is too narrow to observe significant differences. Middle-aged women have consistently been shown to be at increased risk of falls compared with men.^6 31 32^ In our study, female sex was associated with an increased risk of being both intermediate and recurrent falling compared with not falling. Indeed, several studies include only female participants in their samples, reflecting their elevated risk.^4 8 33^

Our findings suggest that recurrent falls in midlife are associated with a broad range of factors, covering not only health-related characteristics but also key socio-demographic and economic indicators.

## Conclusions

A relatively high prevalence of falls and of repeated falls was observed in this longitudinal study of late middle-aged individuals from England. Being a woman, unpartnered, retired or unemployed, lacking home ownership and indicators of poorer health, such as depression, musculoskeletal pain, memory problems and slow walking speed, were all highlighted as important risk factors for recurrent falls. Identifying individuals at increased risk of recurrent falls during midlife is essential as this period represents a critical important window for early detection and intervention before fall risk escalates in later life.

Taken together, these findings underscore the need for fall-prevention strategies in midlife that address not only health-related factors but also the wider social and economic conditions shaping vulnerability. For example, interventions that promote social connectedness, such as community-based group activities, may help mitigate risk among those who are retired or unemployed. Exercise-based programmes, which have proven effective in reducing the rate of falls among community dwelling older adults,^19^ could also be adapted and implemented earlier in the life course. However, the effectiveness of such interventions depends on equitable access and sustained engagement. Evidence suggests that individuals from more disadvantaged backgrounds, who are often at higher risk of falls, are also less likely to engage in interventions to improve health behaviours.^34^ Financial constraints are usually cited as an important barrier to engagement.^35^ Future research should therefore prioritise the development of targeted interventions for accessible physical activity programmes that can reach those most likely to benefit.

## Supplementary material

10.1136/bmjopen-2026-118710 online supplemental file 1

## Data Availability

Data are available upon reasonable request by contacting the corresponding author.
