# Supplementary material for: Risk factors for recurrent falls in midlife: a prospective cohort study using data from the Health and Employment After Fifty (HEAF) study
Source: BMJ Open. 2026 May 27;16(5):e118710. doi: 10.1136/bmjopen-2026-118710 (PMC13218163; doi:10.1136/bmjopen-2026-118710)
Supplement: online supplemental file 1 [file bmjopen-16-5-s001.docx]

**Supplementary Table 1: Multivariate model showing association between selected risk factors and fall categories - bootstrapped CIs**

| **Baseline characteristic** | **Intermediate fallers (vs Non fallers)** | | **Recurrent fallers (vs Non fallers)** | |
| --- | --- | --- | --- | --- |
|  | **RRR (bootstrapped 95%CI)** | **p-value** | **RRR (bootstrapped 95%CI)** | **p-value** |
| Sex, Female | 1.88 (1.69,2.08) | <0.001 | 1.80 (1.42,2.28) | <0.001 |
| Age at recruitment, years | 1.04 (1.03,1.05) | <0.001 | 1.01 (0.97,1.04) | 0.72 |
| Marital status |  |  |  |  |
| Single/widowed/divorced | 1.12 (1.00,1.25) | 0.05 | 1.53 (1.19,1.96) | <0.001 |
| Missing | 1.10 (0.57,2.11) | 0.78 | - |  |
| Qualification level |  |  |  |  |
| No qualification/school only | 0.91 (0.80,1.03) | 0.13 | 0.87 (0.65,1.16) | 0.34 |
| Vocational training certificate | 1.00 (0.88,1.13) | 0.99 | 1.05 (0.78,1.42) | 0.73 |
| University degree or higher | Ref |  | Ref |  |
| Perceived financial status |  |  |  |  |
| At least getting by | Ref |  | Ref |  |
| Managing with difficulty | 0.85 (0.68,1.06) | 0.16 | 0.76 (0.53,1.08) | 0.13 |
| Missing | 0.61 (0.07,5.12) | 0.65 | 0.53 (0.04,7.37) | 0.63 |
| Home ownership |  |  |  |  |
| Rented | 0.91 (0.75,1.10) | 0.35 | 1.05 (0.73,1.52) | 0.79 |
| Mortgaged | 1.04 (0.92,1.18) | 0.50 | 1.37 (1.03,1.82) | 0.03 |
| Owned outright or rent-free | Ref |  | Ref |  |
| Missing | 1.99 (0.24,16.81) | 0.53 | 2.55 (0.20,32.70) | 0.43 |
| Employment status |  |  |  |  |
| Employed | Ref |  | Ref |  |
| Self-employed | 1.03 (0.88,1.22) | 0.70 | 1.83 (0.89,1.99) | 0.17 |
| Unemployed | 1.09 (0.85,1.40) | 0.49 | 2.10 (1.42,3.11) | <0.001 |
| Retired | 0.94 (0.82,1.09) | 0.41 | 1.50 (1.07,2.09) | 0.02 |
| Obesity |  |  |  |  |
| <30 (non-obese) | Ref |  | Ref |  |
| ≥30 (obese) | 1.19 (1.04,1.35) | 0.009 | 1.31 (1.01,1.71) | 0.05 |
| Missing | 1.03 (0.71,1.48) | 0.89 | 1.47 (0.85,2.54) | 0.17 |
| Smoking |  |  |  |  |
| Never/ex | Ref |  | Ref |  |
| Current | 0.90 (0.75,1.07) | 0.22 | 1.18 (0.85,1.65) | 0.31 |
| Missing | 0.68 (0.34,1.37) | 0.28 | 1.15 (0.46,2.89) | 0.76 |
| Self-rated health |  |  |  |  |
| At least good | Ref |  | Ref |  |
| Fair/Poor | 1.35 (1.16,1.56) | <0.001 | 2.40 (1.78,3.24) | <0.001 |
| Missing | 1.22 (0.75,1.98) | 0.43 | 1.32 (0.52,3.70) | 0.51 |
| Depressive symptoms |  |  |  |  |
| Not depressed | Ref |  | Ref |  |
| Depressed | 1.53 (1.34,1.74) | <0.001 | 2.03 (1.55,2.65) | <0.001 |
| Missing | 1.48 (0.85,2.58) | 0.17 | 1.45 (0.49,4.25) | 0.50 |
| Poor sleep |  |  |  |  |
| No | Ref |  | Ref |  |
| Yes | 1.13 (0.97,1.31) | 0.12 | 1.56 (1.18,2.05) | 0.002 |
| Any musculoskeletal pain |  |  | 4.00 (1.41,11.33) | 0.009 |
| No | Ref |  | Ref |  |
| Yes | 1.44 (1.26,1.65) | <0.001 | 2.82 (2.18,3.63) | <0.001 |
| Missing | 0.95 (0.49,1.82) | 0.88 | - |  |
| Walking speed |  |  |  |  |
| Normal/Fast | Ref |  | Ref |  |
| Unable/Very slow | 1.81 (1.33,2.45) | <0.001 | 4.73 (3.29,6.79) | <0.001 |
| Missing | - |  | - |  |
| Problems with your memory |  |  |  |  |
| No serious problems & not worsened | Ref |  | Ref |  |
| Serious problems or a lot worse | 1.34 (0.97,1.84) | 0.08 | 2.02 (1.29,3.17) | 0.002 |
| Missing | 2.31 (1.14,4.69) | 0.02 |  |  |
| CPRD - N. comorbidities |  |  |  |  |
| None | Ref |  | Ref |  |
| At least one | 0.99 (0.88,1.10) | 0.81 | 1.18 (0.87,1.59) | 0.29 |
