# Supplementary material for: Risk factors for recurrent falls in midlife: a prospective cohort study using data from the Health and Employment After Fifty (HEAF) study
Source: BMJ Open. 2026 May 27;16(5):e118710. doi: 10.1136/bmjopen-2026-118710 (PMC13218163; doi:10.1136/bmjopen-2026-118710)
Supplement: Supplementary file 2 [file bmjopen-2026-118710.reviewer_comments.pdf]

PEER REVIEW HISTORY

BMJ Open publishes all reviews undertaken for accepted manuscripts. Reviewers are asked to complete a checklist review form and are provided with free text boxes to elaborate on their assessment. These free text comments are reproduced below.

ARTICLE DETAILS

Title (Provisional)

Risk factors for recurrent falls in midlife: a prospective cohort study using data from the Health and Employment After Fifty (HEAF) study

Authors

D'Angelo, Stefania; Westbury, Leo; Laskou, Faidra; Walker-Bone, Karen; Dennison, E M

VERSION 1 - REVIEW

Reviewer 1

Name Giannarelli, Diana

Affiliation Fondazione Policlinico Universitario A. Gemelli, Facility of Epidemiology and Biostatistics

Date 19-Mar-2026

COI None

I read this paper with interest . Model choice was appropriate and well justified, and the results were reported correctly.

I have a few, mainly minor, points:

1. Were all 7,051 patients followed for 5 years? If not, what was the median follow-up and its range for the entire cohort? This could have affected the appropriate classification of patients.

2. As no training and validation cohorts were used in the model development, the final classification should be verified, at least with a bootstrapping technique

3. In Table 1 why not include a statistical test for age, for example, a Kruskal-Wallis test?

4. In the Methods section, consider mentioning that you followed the STROBE checklist, as you have done

5. Were all the cut-offs used in the analysis predefined in the questionnaire, or were they determined post hoc? In the latter case, please provide a justification for your choices

**Reviewer**	**2**
**Name**	**Sibley , Kathryn**
**Affiliation**	**University of Manitoba, College of Community and Global Health**
**Date**	**28-Mar-2026**
**COI**	**None**

This analysis examined prevalence of falls among 7051 English adults aged 50-64 yrs and explored associations with health, lifestyle, and socioeconomic variables. The overarching premise exploring prevalence of falls in adults across a range of ages has merit, however there are some conceptual gaps in the manuscript that require addressing.

Acknowledging that there are not clear cut offs between aging group categorizations, age-based definitions of groups considered need clear definitions. The authors address these terms and periods with a lack of precision. These include “older adults” (p 4, line 7) and “middle aged adults” (p 4, line 14) and “late middle aged adults” (p 4, line 42).

The statement “Falls among middle-aged adults appear to be relatively common yet remain comparatively understudied” (p 4, lines 13-15) needs referencing.

The authors position the study as population-based, but details supporting this statement are lacking. It’s not clear how the 24 practices were selected, nor how individuals were recruited. As written, this appears to be a convenience sample.

Lack of justification for the socio-economic, lifestyle and health variables tested in the models is the major weakness of the paper. Each variable needs a justification for its inclusion. Some variables tested have been previously established as risk factors in older people, others I struggle to comprehend. This is particularly the case given the number of socio-economic variables that predicted falls. While the authors cannot claim causation, there is some degree of hypothesis for including them, and given how prominently the authors discuss the socio-economic factors in the discussion, the manuscript is missing key details. If the authors cannot appropriately justify all variables, then some should be removed and the analysis revised.

In the results (p 6, line 60), the authors shift their language and start referring to ‘intermediate fallers’. Please revise and be consistent throughout the paper.

Additional limitations about the lack of information on falls could be more fully discussed. Details/ descriptions of fall circumstances would have been highly valued. If younger fallers are falling because they are active or participating in challenging activities, these would be interpreted differently than a fall in a less challenging environment or situation.

The discussion lacks depth and could be enriched. If comments above about socio-economic factors are adequately addressed and this finding holds, there are a number of implications for future work that could be more fully examined. I would focus less on maintaining employment as a protective factor, and more on targeting and increasing access to exercise programs for higher risk groups.

The abstract does not accurately reflect the main text. The abstract emphasizes the health variables, with little mention of the socio-economic variables. The interpretation is quite different than one gets from reading the full manuscript.

VERSION 1 - AUTHOR RESPONSE

Reviewer: 1
Dr. Diana Giannarelli, Fondazione Policlinico Universitario A. Gemelli, IRCCS
Comments to the Author:
I read this paper with interest . Model choice was appropriate and well justified, and the results were reported correctly.

Many thanks for your positive feedback about our manuscript.

I have a few, mainly minor, points:
1. Were all 7,051 patients followed for 5 years? If not, what was the median follow-up and its range for the entire cohort? This could have affected the appropriate classification of patients.

Thank you for this comment. We have now added the information about the number of follow-ups available per person. Please see new text added to the first paragraph of the Results section:

"The number of follow-ups available per person ranged between 1 and 5 (in addition to baseline). 74% of the sample had data on all 5 follow-ups. The remaining 26% was split into: 6% had 4 follow-ups; 7% had 3 follow-ups; 9% had 2 follow-ups and 4% had data on baseline and one follow-up only."

2. As no training and validation cohorts were used in the model development, the final classification should be verified, at least with a bootstrapping technique

Thank you for your comment. We have re-run the final multivariate model with bootstrap resampling (1000 reps) and added a Table in the Supplementary material showing estimates and bootstrapped confidence intervals. We were reassured to see that bootstrapped confidence intervals were similar to the ones reported in the manuscript. We have added the following sentence to the statistical analysis section of the paper.

“Internal validation of the final multivariate model was performed using bootstrap resampling with 1,000 replications. Estimates with bootstrapped confidence intervals are reported in Supplementary Table 1.”

3. In Table 1 why not include a statistical test for age, for example, a Kruskal-Wallis test?

Thank you for your comment. Apologies for not including a p-value for this baseline characteristic, this was an oversight. This is now provided in Table 1.

4. In the Methods section, consider mentioning that you followed the STROBE checklist, as you have done

Many thanks for the helpful comment. We have added mentioning of the STROBE checklist to the Methods.

“The reporting of this observational study follows the STROBE checklist.”

5. Were all the cut-offs used in the analysis predefined in the questionnaire, or were they determined post hoc? In the latter case, please provide a justification for your choices

Thank you for your comment. The questionnaire included mostly categorical variables, however, several categories contained very small numbers. To retain statistical power and avoid sparse data issues, we have re-categorised some variables prior to analysis. Wherever possible, we applied cut-offs that had already been used in previous publications based on the same cohort. Please find new text below:

“Most variables were re-categorised prior to the analysis to increase statistical power and avoid sparse data issues.”

Reviewer: 2
Dr. kathryn Sibley , University of Manitoba
Comments to the Author:
This analysis examined prevalence of falls among 7051 English adults aged 50-64 yrs and explored associations with health, lifestyle, and socioeconomic variables. The overarching premise exploring prevalence of falls in adults across a range of ages has merit, however there are some conceptual gaps in the manuscript that require addressing.

Acknowledging that there are not clear cut offs between aging group categorizations, age-based definitions of groups considered need clear definitions. The authors address

these terms and periods with a lack of precision. These include “older adults” (p 4, line 7) and “middle aged adults” (p 4, line 14) and “late middle aged adults” (p 4, line 42).

Thank you for your helpful comment. We have tightened our language and made sure that “late middle-aged adults” is used throughout the manuscript.

The statement “Falls among middle-aged adults appear to be relatively common yet remain comparatively understudied” (p 4, lines 13-15) needs referencing.

Thank you, this was an oversight. We have added references in support of this sentence.

The authors position the study as population-based, but details supporting this statement are lacking. It’s not clear how the 24 practices were selected, nor how individuals were recruited. As written, this appears to be a convenience sample.

Thank you for this comment and for the opportunity to clarify the recruitment process. We have added details of the recruitment process to the first paragraph of the Methods. Please find next text below:

“Participants were recruited from 24 general practice surgeries, chosen among those who contributed data to the Clinical Practice Research Datalink (CPRD). The surgeries mailed a consent form and a baseline questionnaire to all individuals born between 1948 and 1964 (other than those suffering from terminal illnesses or recently bereaved). Those who returned a written consent form and a completed baseline questionnaire to the HEAF research team, were enrolled into the study and were sent annual follow-up questionnaires.”

Lack of justification for the socio-economic, lifestyle and health variables tested in the models is the major weakness of the paper. Each variable needs a justification for its inclusion. Some variables tested have been previously established as risk factors in older people, others I struggle to comprehend. This is particularly the case given the number of socio-economic variables that predicted falls. While the authors cannot claim causation, there is some degree of hypothesis for including them, and given how prominently the authors discuss the socio-economic factors in the discussion, the manuscript is missing key details. If the authors cannot appropriately justify all variables, then some should be removed and the analysis revised.

Thank you for this comment. The aim of the paper was to explore which variables were the strongest predictors of intermediate and recurrent falls. We agree with the reviewer that the rationale for inclusion of these factors should be clarified and we have

expanded the "Methods" section accordingly by adding relevant references to justify our choice of variables.

"Because falls are multifactorial events, influenced by socio-demographic, lifestyle, physical and behavioural factors, we selected variables across each of these domains.

Socio-demographic factors – including age, sex, highest educational qualification, marital status (single/widowed/divorced, married/living with partner) [13, 14], as well as indicators of socioeconomic position such as home ownership (rented, mortgaged, owned outright/rent free), self-perceived financial status (at least getting by financially, managing with difficulty), proportion of family income contributed by the participant (≥1/2, between 1/2 and 1/4, less than 1/4) [15, 16], and employment status (employed, self-employed, unemployed, retired) are widely recognised as important determinants of falls. These factors influence exposure to environmental hazards, access to resources and overall vulnerability to adverse health outcomes, including recurrent falls.

Lifestyle factors such as living with obesity (BMI was calculated based on self-reported height and weight and obesity was present if BMI≥30 kg/m2) [17], alcohol consumption [18], smoking habits, and leisure time physical activity (none, ≤5 hours/week, 5-10 hours/week, >10 hours/week) [19] can influence balance, mobility and exposure to activity-related hazards. Health-related variables like self-rated health (SRH) (fair/poor, at least good), depressive symptoms assessed with the CES-D score (coded as depressed if CES-D≥16), poor sleep, any musculoskeletal pain in the previous 12 months, walking speed (normal/fast vs very slow/unable to walk), and memory problems (no serious problems and not worsened in the past year vs serious problems or a lot worse in the past year) capture functional and clinical characteristics that may indicate underlying health impairments [20-22]. Most variables were re-categorised prior to the analysis to increase statistical power and avoid sparse data issues."

In the results (p 6, line 60), the authors shift their language and start referring to 'intermediate fallers'. Please revise and be consistent throughout the paper.

Thank you for this comment. However, we are unsure about what the reviewer is referring to. The outcome of this manuscript is a three-category variable defined as follows: non-fallers, intermediate fallers, and recurrent fallers. In the Results section we start by presenting estimates that relate to recurrent fallers (vs non-fallers) and then move on to describe effect estimates for the category of intermediate fallers (vs non-fallers). We hope this in now clearer to the reviewer.

Additional limitations about the lack of information on falls could be more fully discussed. Details/ descriptions of fall circumstances would have been highly valued. If younger fallers are falling because they are active or participating in challenging activities, these would be interpreted differently than a fall in a less challenging environment or situation.

Thank you for the suggestion. We agree with the reviewer that these data would have been helpful, however they were not available in this dataset. We have expanded the section on the limitations of the study around the outcome definition as detailed below:

"Furthermore, information on fall severity, consequences, whether the fall resulted in hospitalisation, and the circumstances surrounding each fall was not collected. The lack of contextual detail limits our ability to distinguish between falls occurring during high-intensity activities and those taking place during lower intensity activities."

The discussion lacks depth and could be enriched. If comments above about socio-economic factors are adequately addressed and this finding holds, there are a number of implications for future work that could be more fully examined. I would focus less on maintaining employment as a protective factor, and more on targeting and increasing access to exercise programs for higher risk groups.

Thank you for your comment. We have expanded the Conclusions section suggesting potential future research. Please find the new text below:

"However, the effectiveness of such interventions depends on equitable access and sustained engagement. Evidence suggests that individuals from more disadvantaged backgrounds, who are often at higher risk of falls, are less likely to engage in interventions to improve health behaviours [25]. Financial constraints are usually cited as an important barrier to engagement [26]. Future research should therefore prioritise the development of targeted interventions for accessible physical activity programmes that can reach those most likely to benefit."

The abstract does not accurately reflect the main text. The abstract emphasizes the health variables, with little mention of the socio-economic variables. The interpretation is quite different than one gets from reading the full manuscript.

Thank you for the comment. We have amended the conclusion section in the abstract to highlight the importance of socio-economic risk factors.

“Recurrent falls in mid-life were relatively common. Both socio-economic, and health-related characteristics, alongside female gender, were identified as predictors, suggesting potential targets for early identification and risk mitigation in this age group.”

VERSION 2 - REVIEW

**Reviewer**	**1**
**Name**	**Giannarelli, Diana**
**Affiliation**	**Fondazione Policlinico Universitario A. Gemelli, Facility of Epidemiology and Biostatistics**
**Date**	**05-May-2026**
**COI**	

Thank you for answering all my questions.

**Reviewer**	**2**
**Name**	**Sibley , Kathryn**
**Affiliation**	**University of Manitoba, College of Community and Global Health**
**Date**	**06-May-2026**
**COI**	

Thank you to the authors for addressing my comments. My only outstanding comment relates to the "intermediate faller" label that is introduced in the data analysis section and continues into the results, which is still unclear. Does an “intermediate faller” refer to a person who had a single fall? (in contrast to a “non-faller”, or a “recurrent faller”?) If yes, could this please be revised to ‘single fallers’ (or something equivalent) to help with readability. If no, could “intermediate faller” be defined? Thank you.

VERSION 2 - AUTHOR RESPONSE

Reviewer: 1
Dr. Diana Giannarelli, Fondazione Policlinico Universitario A. Gemelli, IRCCS
Comments to the Author:
Thank you for answering all my questions.

Thank you for the time taken to review our paper.

Reviewer: 2
Dr. Kathryn Sibley, University of Manitoba
Comments to the Author:
Thank you to the authors for addressing my comments. My only outstanding comment relates to the "intermediate faller" label that is introduced in the data analysis section and continues into the results, which is still unclear. Does an “intermediate faller” refer to a person who had a single fall? (in contrast to a “non-faller”, or a “recurrent faller”?) If yes, could this please be revised to ‘single fallers’ (or something equivalent) to help with readability. If no, could “intermediate faller” be defined? Thank you.

Thank you and we apologise if this definition was not sufficiently clear. In our study, the category of “intermediate fallers” refers to participants who reported at least one fall during follow-up but did not meet the criteria for recurrent falling. Specifically, these individuals did not experience *more than one fall on at least two occasions* (the definition of recurrent fallers), nor did they remain fall-free throughout the study (non-fallers).

Please find below the amended text, in the Methods section:

“Participants were categorised as recurrent fallers if they experienced more than one fall on at least two occasions over the course of the study; non-fallers if they never reported a fall over the study duration, and intermediate fallers if they reported at least one fall but did not meet the criteria for recurrent fallers.”
